# Advances in controlled release drug delivery systems based on nanomaterials in lung cancer therapy: A review

**DOI:** 10.1097/MD.0000000000041415

**Published:** 2025-02-07

**Authors:** Jiang Fu, Li Yu, Zixu Wang, Haoyu Chen, Song Zhang, Haining Zhou

**Affiliations:** a Department of Thoracic Surgery, Suining Central Hospital, Suining, China; b School of Medical and Life Science, Chengdu University of Traditional Chinese Medicine, Chengdu, China; c Department of Physical Examination, Suining Central Hospital, Suining, China; d National Center for Translational Medicine (Shanghai) SHU Branch, Shanghai University, Shanghai, China

**Keywords:** drug delivery systems, lung cancer, nanocarriers, nanotechnology, precision therapy

## Abstract

Lung cancer is one of the most common malignant tumors, with the highest morbidity and mortality rates. Currently, significant progress has been made in the treatment of lung cancer, which has effectively improved the overall prognosis of patients, but there are still many problems, such as tumor recurrence, drug resistance, and serious complications. With the rapid development of nanotechnology in the field of medicine, it breaks through the inherent limitations of traditional cancer treatments and shows great potential in tumor treatment. To address the drawbacks of traditional therapeutic means, nanodrug delivery systems can release drugs under specific conditions, thus realizing tumor-targeted drug delivery, which improves the antitumor effect of drugs. In this paper, we review the current treatments for lung cancer and further discuss the advantages and common carriers of nanodrug delivery systems. We also summarize the latest research progress of nanotargeted drug delivery systems in the field of lung cancer therapy, discuss the problems faced in their clinical translation, and look forward to future development opportunities and directions.

## 1. Introduction

**Figure 1.**
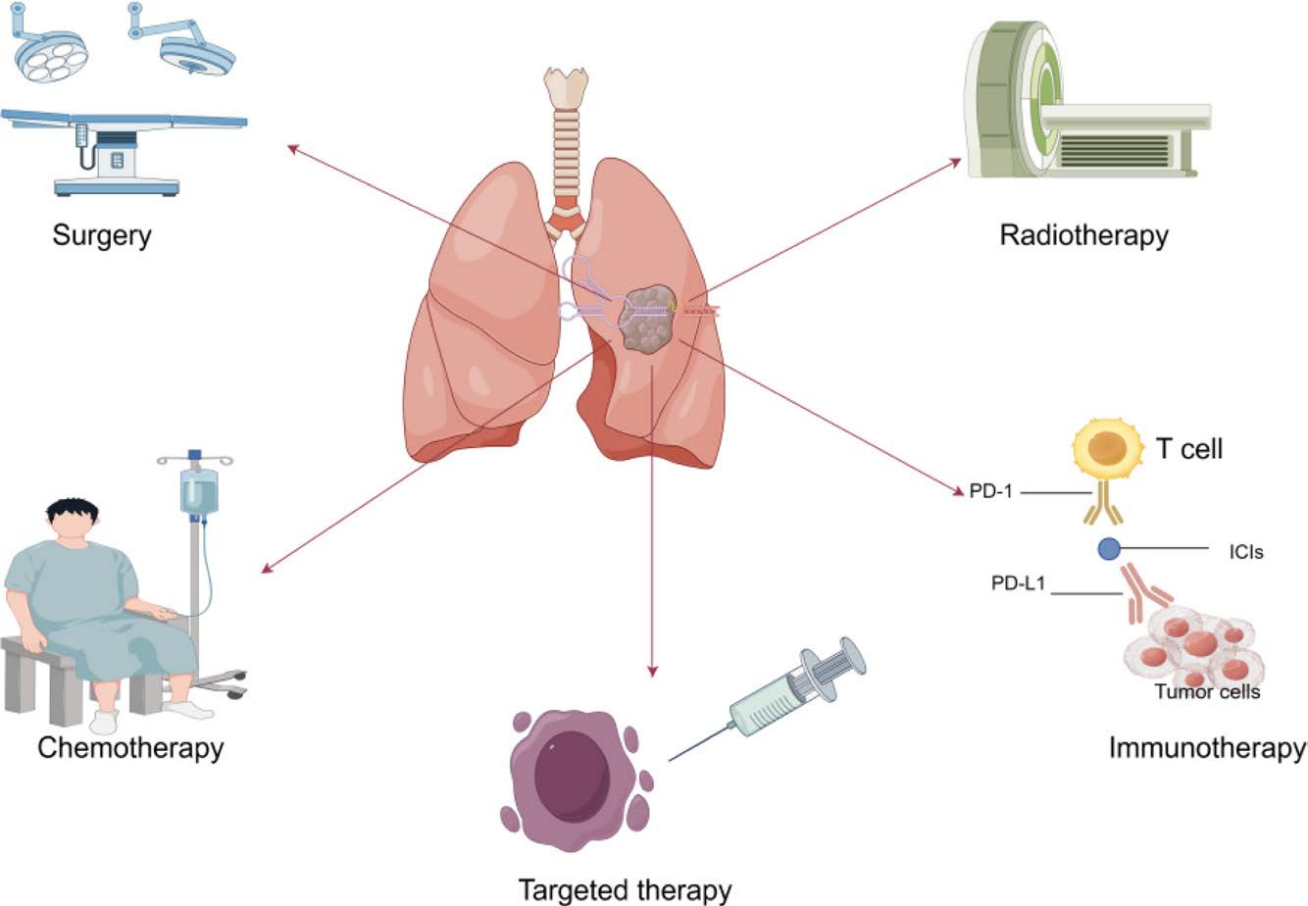
The current primary treatment modalities for lung cancer. PD-1 = programmed cell death receptor 1, PD-L1 = programmed cell death ligand 1.

Lung cancer is the most common malignant tumor worldwide, with approximately 2.5 million new lung cancer cases in 2022. It ranks first in morbidity and mortality among all diagnosed malignant tumors, and thus lung cancer is still the leading cause of cancer-related deaths.^[1]^ According to histopathology, lung cancer can be divided into small cell lung cancer (SCLC) and non-small cell lung cancer (NSCLC), and the incidence of NSCLC accounts for about 85% of lung cancer. NSCLC is mainly categorized into squamous cell carcinoma, adenocarcinoma, and large cell carcinoma.^[2]^ NSCLC can choose different treatment modalities according to the clinical stage (Fig. 1), mainly including surgery, chemotherapy, radiotherapy, targeted therapy, and immunotherapy.^[3]^ Due to the lack of specific tumor markers and clinical symptoms in the early stage, most lung cancer patients have already metastasized when they are diagnosed, which brings great difficulties in treatment.^[4]^ Although the treatment of lung cancer has progressed very rapidly in recent decades, the survival rate of lung cancer patients is still low.^[5]^ Therefore, there is an urgent need for a new strategy to treat lung cancer and improve the quality of life.

Nanotechnology, as an emerging technology, has a wide impact in the social sciences and biomedical fields. In recent years, nanotechnology has been utilized in the treatment and diagnosis of many diseases, such as cancer and cardiovascular diseases.^[6]^ Nanomaterials are used as drug carriers due to their small size, large specific surface area, and high permeability. Moreover, nanomaterials have physicochemical properties such as acoustic, optical, electrical, magnetic, and thermal properties that enable controlled release of drugs. Nanodrug delivery systems (NDDS) are drug delivery systems consisting of nanomaterials with a diameter of <1000 nm.^[7]^ NDDS can place drugs on the surface or inside nanomaterials by encapsulation, adsorption, polymerization, and coupling, thus preventing drug degradation and delivering drugs accurately to tumor tissues.^[8]^ Therefore, NDDS have the functions of efficient drug loading, targeted delivery, and controlled drug release in tumor therapy.^[9]^

In this review, we briefly describe the current treatments for lung cancer and their drawbacks and further summarize the advantages of drug delivery systems and common carriers. Finally, we also summarize the recent advances of NDDS in surgery, chemotherapy, radiotherapy, immunotherapy, and targeted therapy for lung cancer and the problems they face in clinical translation.

## 2. Lung cancer treatments and their disadvantages

Surgery is still the primary treatment for early stage lung cancer and can improve patient survival.^[10]^ However, incomplete removal of the tumor may lead to recurrence or distant metastasis. Excessive resection of lung tissue can reduce the patient’s lung function, which can affect the patient’s quality of life.^[11]^ Most lung cancer patients are diagnosed at an advanced stage and lose the opportunity for surgery. Although chemotherapy can prolong the survival of patients with advanced lung cancer, it often causes side effects such as gastrointestinal reactions, liver function abnormalities, and allergic skin reactions.^[12]^ Additionally, with the circulation and metabolism of the drug in the body, it is difficult to control the time and concentration of the drug to reach the tumor site, which ultimately leads to the failure of chemotherapy. With the rapid development of imaging technology, the role of radiotherapy in the treatment of lung cancer has become increasingly prominent. However, radiotherapy also faces several challenges, such as increased tumor resistance to radiotherapy and damage to surrounding healthy tissue from ionizing radiation.^[13,14]^ Therefore, increasing the sensitivity of tumor tissue to radiotherapy can help to enhance the efficacy of radiotherapy. Immunotherapy for tumors has developed very rapidly in recent years. However, the efficacy of immunotherapy is limited by the presence of the tumor microenvironment (TME).^[15]^ In addition, most immunotherapeutic drugs, such as peptides and monoclonal antibodies, are biomolecules, and how to deliver these drugs to tumor tissues and maintain their activity to exert their therapeutic effects is also a problem that needs to be solved in immunotherapy. With the rapid development of genetic testing technology, molecularly targeted therapy has become an important treatment for advanced lung adenocarcinoma. Compared with ordinary chemotherapeutic drugs, targeted therapy has the advantages of stronger targeting, lower toxic side effects, and better efficacy.^[16]^ Epidermal growth factor receptor (EGFR) and anaplastic lymphoma kinase are the most important targets for molecularly targeted therapy in lung cancer.^[17]^ Tyrosine kinase inhibitors (such as gefitinib [GEB] and ositinib) have been developed to target EGFR mutations, which have dramatically prolonged patient survival.^[18]^ However, acquired resistance is often unavoidable due to acquired T790M mutations, tumor heterogeneity, and off-target side effects, severely impacting treatment outcomes.^[19]^

## 3. Advantages of NDDS

As nanotechnology and nanomedicine develop rapidly, NDDS has received widespread attention as a strategy to improve drug delivery efficiency and tumor therapy. Compared with traditional therapeutic methods, drug delivery systems using nanomaterials as carriers have the following advantages: improve the solubility of hydrophobic drugs, thus reducing the amount of drugs used and improving the efficacy of drugs; achieve precision treatment of tumors through active or passive targeting; and reduce the hindering effect of physiological barriers on drugs and prolong blood circulation time.

### 3.1. Improving the therapeutic effect of drugs

Nanomaterials feature small sizes, large specific surfaces, and easy modification, which enable them to encapsulate, adsorb, or covalently cross-link hydrophobic drug molecules so that the chemotherapeutic drugs can form a high local concentration in the carrier.^[20,21]^ Studies have shown that the combination of paclitaxel (PTX) with albumin improves the water solubility of PTX and reduces the incidence of allergic reactions.^[22]^ In addition, by providing stable and hidden storage space for drug molecules, nanomaterials can prevent degradation and inactivation during the delivery of drugs into the human body, thus maintaining the activity of drugs.^[23]^ This is beneficial to increase the concentration of anticancer drugs in the lesion site, reduce the number of times of drug delivery, and improve the effect of tumor treatment. Moreover, the same nanocarrier can be loaded with 2 or more kinds of drugs to achieve synergistic and efficient therapeutic purposes.^[24]^ It was found that PEGylated liposomes were loaded with PTX and carboplatin to prepare nanoparticles, and the tumor inhibition rate of the nanoparticles was 2.07 times that of the PTX/carboplatin treatment group, which greatly improved the therapeutic effect.^[25]^

### 3.2. Achieving precision treatment of tumors

The targeted delivery of NDDS includes both passive targeting and active targeting.^[26]^ In normal conditions, the nanocarriers cannot pass through the vascular endothelial cells to enter the normal tissues; however, there are gaps between the endothelial cells of neovascularization in the tumor tissues, so the nanocarriers can penetrate into the tumor tissues during the blood circulation. In addition, the number of lymphatic vessels in the tumor tissue is small, and the lymphatic reflux is blocked, so the nanodrugs retained in the tumor tissue cannot be removed in time, which further promotes the enrichment of nanodrugs in the tumor site. This phenomenon is known as the enhanced permeability and retention effect (EPR effect).^[27]^ The transport of nanocarriers to the tumor region through leaky blood vessels is known as passive targeting.^[28]^ Passive targeting is usually achieved through the EPR effect but also depends on the physicochemical properties of the nanocarriers (such as shape and size) and important characteristics of the cancer cells (such as temperature, pH, and surface charge of tumor cells).^[29]^ Passive targeting allows nanodrugs to be enriched in tumor tissues, thereby increasing their antitumor efficacy and reducing their side effects. However, when the nanocarriers enter the blood, serum proteins may bind to the surface of the nanocarriers, resulting in the formation of a protein corona.^[30]^ It has been found that the protein corona’s size, diameter, and surface properties also influence passive targeting.^[31]^ Therefore, the surface of the nanocarriers may need to be modified by polymers, stabilizers, or proteins to enhance their affinity for cells and reduce protein corona formation.

In addition to their unique size effect, nanocarriers can also be functionally modified to achieve targeted aggregation of tumor tissues and reduce the toxic side effects on normal tissues and cells. Compared with normal cells, the expression of some receptors on the surface of tumor cells is increased. Active targeting is to connect corresponding ligands, such as peptides, proteins, and antibodies, to the nanocarriers, which can specifically recognize the overexpressed receptors on the surface of the tumor cells and thus deliver the drugs to the TME in an actively targeted manner.^[32]^ Common targets currently used for active targeting of lung cancer include EGFR, vascular endothelial growth factor, folate receptor, and transferrin receptor.^[33]^ By utilizing various ligands to specifically bind to these receptors on the surface of lung cancer cells and designing nanocarrier systems for effective targeting, targeted drug delivery can be achieved, thus reducing the incidence of side effects.

### 3.3. Prolonging the blood circulation of drugs

As mentioned earlier, nanocarriers have the ability to deliver drugs to tumor tissues. However, the biological barrier during drug delivery prevents the accumulation of drugs in tumor tissues, thus limiting the efficacy of the drugs. For example, after the drugs enter the blood circulation through intravenous administration, the reticulocytes of the liver, spleen, and other organs and the endothelial cells of the blood vessels can phagocytose the foreign substances in the body with the help of pseudopods, and these phagocytotic cells are collectively known as the mononuclear phagocyte system (MPS), also known as the reticuloendothelial system (RES).^[34]^ Polyethylene glycol modification is one of the classical strategies to overcome physiological barriers.^[35]^ For example, polyethylene glycol modification on the surface of liposomes can be effective in avoiding recognition and uptake by RES, thus prolonging blood circulation time.^[36]^ In addition, biomimetic nanoparticles, including cell membranes, extracellular vesicles, and viruses, can prolong the in vivo circulation of nanocarriers by mimicking physiological processes or modes of action in the human body and by avoiding direct removal by the RES.^[37]^ Thus, NDDS can reduce the obstruction of physiological barriers and increase the concentration of drugs in tumor tissues.

## 4. Carriers for nanomedicine delivery systems

**Figure 2.**
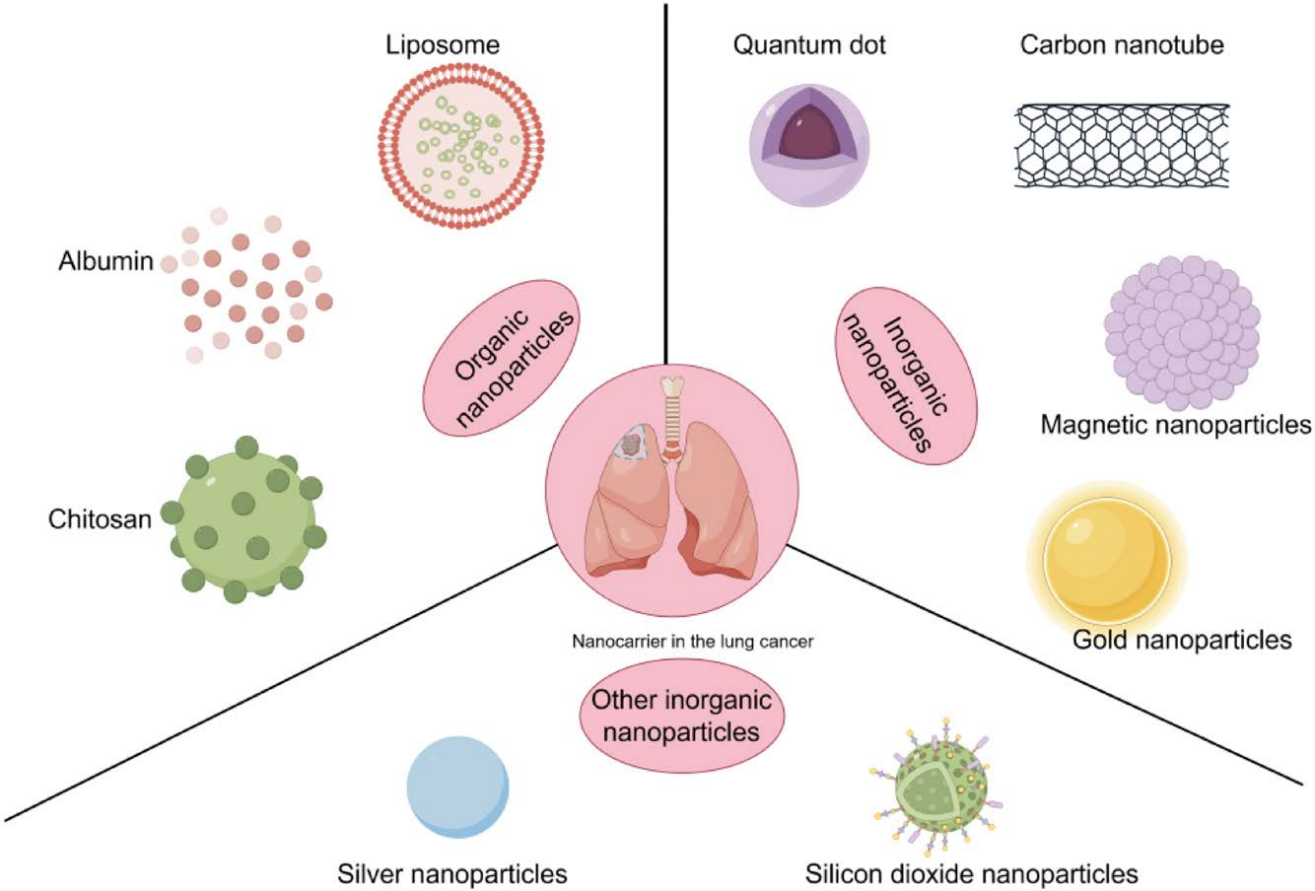
Classification of carriers for nanodrug delivery systems.

The types of carriers for NDDS can be categorized into organic and inorganic nanomaterials based on the properties of the nanomaterials (Fig. 2). Organic nanomaterials mainly include liposomes, proteins, and chitosan. Inorganic nanomaterials mainly include carbon nanomaterials, magnetic nanomaterials, gold nanomaterials, and other metallic and nonmetallic nanomaterials. These nanocarriers show great potential to improve the performance of existing drugs, reduce their systemic side effects, and enhance therapeutic efficacy in lung cancer treatment. Table 1 summarizes the clinical trials of different nanocarriers in lung cancer.

**Table 1:** Nanocarriers that have undergone clinical trials with lung cancer patients.

Formulation/drug	Nanocarrier	Stage/disease	Phase	Status	Trial ID	Primary outcome
LY01610 (irinotecan hydrochloride liposome Injection)	Liposome	SCLC	III	Recruiting	NCT06128837	Evaluating OS
FF-10850 (topotecan liposome injection)	Liposome	SCLC	I	Recruiting	NCT04047251	Evaluating MTD and DLT
Irinotecan liposome injection (ONIVYDE®)	Liposome	SCLC	III	Completed	NCT03088813	Evaluating OS and DLT
Abraxane (paclitaxel Albumin-Stabilized Nanoparticle)	Albumin nanoparticles	IV NSCLC	II	Completed	NCT01620190	Evaluating CR and RR
Carboplatin and nab paclitaxel	Albumin nanoparticle	IIIB/IIIC/IV NSCLC	III	Active, not recruiting	NCT04033354	Evaluating PFS
Paclitaxel ALB-stabilized nanoparticle	Albumin nanoparticle	IIIB/IV NSCLC	II	Completed	NCT00729612	Evaluating CR and PR
Paclitaxel albumin-stabilized nanoparticle	Albumin nanoparticle	IV NSCLC	I/II	Completed	NCT00077246	Evaluating MTD and DLT
ABI-009 (human albumin-bound rapamycin)	Albumin nanoparticle	NSCLC	II	Terminated	NCT03670030	Evaluating CR and PR
Hafnium Oxide-containing nanoparticles NBTXR3	Nanoparticle	I–III NSCLC	I	Recruiting	NCT04505267	Evaluating MTD and DLT

CR = complete response, DLT = dose-limiting toxicities, MTD = maximum tolerated dose, NSCLC = non-small cell lung cancer, OS = overall survival, PFS = progression-free survival, RR = partial response, SCLC = small-cell lung cancer.

Source: https://clinicaltrials.gov/

### 4.1. Organic nanomaterials

#### 4.1.1. Liposome

Liposomes are spherical nanovesicles formed by an aqueous core surrounded by 1 or more hydrophobic lipid bilayers. The liposome membrane structure is mainly composed of phospholipids and cholesterol. Due to the amphiphilic affinity of phospholipids, liposomes mimic biological cell membranes well, thus promoting cellular uptake.^[38]^ The size of liposomes is usually between 20 nm and several microns, and its size mainly depends on the lipid composition and the number of bilayers.^[39]^ With a hydrophilic core and a hydrophobic shell, liposomes can encapsulate both hydrophilic and hydrophobic drugs.^[40]^ By encapsulating the drug, liposomes can prevent the drug from being affected by the physiological environment and avoid toxic effects on normal tissues; therefore, liposomes are widely used to deliver a wide range of anticancer drugs in lung cancer therapy.

Several related studies have shown that liposomes can improve drug utilization and stability and reduce toxic side effects in vivo. For example, Hu et al encapsulated GEB in liposomes to prepare nanoliposomal compound drugs (GL). The results showed that GL could greatly delay the release of the drug and prolong its duration of action compared with GEB alone. In addition, GL inhibited the migration and invasion of A549 cells and promoted the apoptosis of A549 cells, which significantly improved the therapeutic effect.^[41]^ Since liposomes can be rapidly absorbed by RES, many researchers have further modified the surface of nanoliposomes by polyethylene glycolization to improve the hydrophilicity of the materials and to further prolong the duration of liposomal drugs in the blood circulation.^[42]^ It was demonstrated that nedaplatin (NDP) was encapsulated in polyethylene glycolized liposomes to prepare compound drugs (NDP-LPs), and the release time of NDP could reach up to 8 days. In addition, the NDP-LPs could accumulate in lung cancer tissues, which greatly inhibited the growth of tumors.^[43]^

In the treatment of malignant tumors, a number of liposomal nanodrugs have been successfully marketed through clinical trials and are widely used in the clinic. For example, irinotecan liposome for metastatic pancreatic cancer, adriamycin liposome for breast cancer, and PTX liposome for NSCLC. However, liposomes also face many challenges; for example, the tumor extracellular matrix restricts the penetration of liposome carriers from the tumor vasculature into the surrounding space, which ultimately leads to a limited amount of drug release from the carriers.^[44]^ In addition, one of the keys is how to control the rate of drug release in the lesion to avoid the development of drug resistance in tumor cells. In summary, future research and development of liposomal NDDS will focus on targeting and responding to internal and external stimuli (such as temperature, ultrasound, and enzymes) to achieve precision tumor therapy.

#### 4.1.2. Protein nanomaterials

Protein nanomaterials have the advantages of biodegradability, biocompatibility, low immunogenicity, and stability. Protein nanomaterials commonly used in tumor therapy include albumin, whey proteins, lipoproteins, and silk proteins. PTX albumin nanoparticles (Abraxane) developed by Celgene use human serum albumin as a carrier, which can be rapidly distributed and aggregated in tumors through the natural transport pathway of albumin (albumin binds to the receptor proteins and the foveal proteins) in vivo to improve the efficacy and reduce the toxicity of chemotherapy.^[45]^ KRAS is the most common mutated gene in human cancers. Nanoparticles prepared with bovine serum albumin as a carrier can deliver siRNA targeting the KRAS G12S mutation, which avoids the interpretation of siRNA by nuclease during the delivery process and thus improves the transfection efficiency of siRNA on lung cancer cells.^[46]^ Jayaprakasha et al^[47]^ prepared curcumin-whey protein nanoparticles by the desolventization method, which showed a significant increase in uptake in colon and prostate cancers as compared to free curcumin. Wu et al^[48]^ prepared PTX-silk protein nanoparticles by self-assembly at room temperature for the local treatment of gastric cancer, and the nanoparticles were specifically taken up by human gastric cancer cells and significantly inhibited tumor development.

It can be seen that albumin has the widest application in drug delivery. In addition to the marketed PTX albumin nanopreparations, other types of albumin nanopreparations are also undergoing extensive research.^[49]^ How to simplify the preparation method, save costs, and improve the safety and targeting of protein nanoformulations are future research directions.

#### 4.1.3. Chitosan nanomaterials

Chitosan is a natural multifunctional polysaccharide with good biocompatibility and biodegradability, but poor solubility limits its usefulness in the biomedical field. The solubility of chitosan is altered by introducing different functional groups to the surface of chitosan through chemical reactions such as acylation, alcoholization, and amination.^[50]^ Chitosan has been widely used as a nonviral delivery carrier for drugs. Chitosan nanoparticles (ChNPs) not only affect the metabolism of tumor cells by inducing apoptosis and inhibiting cell growth but also effectively inhibit the proliferation of tumor cells by increasing the concentration of drugs in tumor tissues.^[51]^ Amin et al^[52]^ prepared chitosan nanoparticles (Gef-CSNP) of GEF and demonstrated that it was more toxic than a single Gef to human NSCLC A549 cells by cellular experiments. In 1 study, chitosan nanoparticles (FA-CS-SB-NPs) were prepared using folic acid (FA) and silibinin (SB), and FA-CS-SB-NPs had good slow-release properties, which improved the therapeutic efficacy of SB in lung cancer.^[53]^ Zhu et al^[54]^ developed novel T7 peptide-modified nanoparticles (CBT-DC) based on carboxymethyl chitosan for codelivering docetaxel and curcumin to human lung cancer cells and found that CBT-DC could better improve the immunosuppressive microenvironment and significantly inhibit the proliferation of lung cancer as compared with docetaxel and curcumin treatment alone.

At present, most of the studies on ChNPs in tumor therapy remain at the cellular and animal levels, with little clinical research. In basic research, ChNPs also have some problems, for example, how to effectively improve their targeting, increase their stability in the blood, and increase the concentration of drugs in tumor tissues. In order to solve the above problems and apply ChNPs in the clinic, we need to make continuous efforts to develop ChNPs with stronger efficacy, better targeting, higher stability, and better safety.

### 4.2. Inorganic nanomaterials

#### 4.2.1. Carbon nanomaterials

Carbon nanomaterials are promising emerging therapeutic tools and carriers with low toxicity, good biocompatibility, unique optical properties, a large specific surface area, and the ability to accumulate in tumors through the EPR effect. In recent years, carbon nanomaterials have been widely used in tumor therapy. Carbon nanomaterials commonly used in the treatment of lung cancer include quantum dots (QDs), carbon nanotubes (CNTs), graphene oxide (GO), and mesoporous carbon nanospheres.

QDs are typically made from semiconductor materials of periodic groups II–VI or III–V and have unique optical properties.^[55]^ Researchers coupled desmethyl erlotinib to QDs, which improves drug delivery efficiency in vivo and enhances erlotinib’s toxicity to lung cancer cells.^[56]^ CNTs are cylindrical, hydrophobic tubes composed of carbon atoms. According to the number of graphene layers, nanotubes are categorized as single-walled nanotubes and multi-walled nanotubes.^[26]^ Doxorubicin (DOX) and CNT are bonded by covalent bonding. The concentration of this complex (CNT-DOX) can be maintained for a long period of time in multidrug-resistant lung cancer cells and significantly inhibits the proliferation of drug-resistant lung cancer cells.^[57]^ Kamazani et al coupled bromocriptine with a novel multi-walled CNT. This complex was highly toxic to lung cancer A549 cells but not to normal cells, suggesting that it is highly selective for the treatment of lung cancer.^[58]^ The GO surface consists of oxygenated hydrophilic groups. It is a suitable candidate for drug delivery system applications due to its better biocompatibility. Reduction of GO to RGO by thermal, chemical, photochemical, electrochemical, or green reduction methods helps to preserve the characteristics of graphene.^[59]^ Wei et al loaded gemcitabine on reduced graphene oxide (GEM-rGO). In in vivo and in vitro experiments, GEM-rGO significantly inhibited the growth of lung cancer; moreover, due to its strong near-infrared (NIR) absorption, it could further enhance the photothermal ablation of tumors and realize the combined treatment of tumors.^[60]^

In terms of biosafety, QDs and mesoporous carbon nanospheres have higher biosafety, with no obvious toxicity at higher concentrations after modification. In addition, further exploration of carbon nanomaterials for tumor-targeting and surface modification technologies will remain a research focus in this field.

#### 4.2.2. Magnetic nanomaterials

Magnetic nanoparticles (MNPs) are generally magnetic composites composed of iron, nickel, cobalt, and other metals and their oxides. With a large specific surface area and magneto-thermal effect, MNPs are easy to enrich and separate, which has a broad application prospect in tumor therapy.^[61]^ Fang et al prepared PPy@Fe_3_O_4_NPs by the electrostatic adsorption method, and the photothermal effect generated by irradiation with NIR light can effectively destroy tumor cells. In addition, PPy@Fe_3_O_4_NPs can generate toxic hydroxyl radicals (·OH); therefore, PPy@Fe3O4NPs can inhibit tumor growth and metastasis through the combination of chemodynamic therapy and photothermal therapy (PTT). It was demonstrated by in vitro cellular experiments that PPy@Fe_3_O_4_NPs inhibited the migration and induced apoptosis of A549 cells. In a nude mouse model, mice injected with PPy@Fe_3_O_4_NPs showed a significant reduction in tumor volume and body weight compared with the control group.^[62]^

Although significant progress has been made in the application of MNPs in the field of tumor therapy, there are still some problems, such as interference with DNA synthesis and cardiotoxicity.^[63]^ In addition, how to simplify the preparation process, reduce the cost, and understand their metabolism and distribution in vivo are also issues that need to be addressed. Therefore, the clinical application of this new material still faces challenges.

#### 4.2.3. Gold nanomaterials

Gold nanomaterials are characterized by low toxicity, good biocompatibility, special optical properties, and a large specific surface area, and gold nanoparticles of various shapes and sizes can be synthesized by chemical, physical, or biological methods.^[64]^ The most widely used in tumor therapy are gold nanorods, followed by gold nanocages. Due to their unique photothermal effect, gold nanoparticles can be used for photodynamic therapy (PDT), PTT, and drug therapy to achieve synergistic therapeutic effects on lung cancer at the same time.

The researchers utilized gold nanorods to synthesize a nanocomplex (AuNR@S-MCM-41) with Mobil Composition of Matter No. 41 (MCM-41), which is capable of significantly warming up under irradiation of NIR light to improve the photothermal efficiency against cancer cells. In addition, loading AuNR@S-MCM-41 with the anticancer drug DOX yielded AuNR@S-MCM-41-DOX, which exhibited excellent efficacy in killing A549 cells by combining PTT and chemotherapy compared with single chemotherapy or PTT.^[65]^ Chen et al prepared nanocomplexes (B7H3/Dox@GNCs) by combining B7 homologue 3 protein (B7-H3), gold nanocages, and Dox, which improved the stability of Dox in circulation. It was found that B7H3/Dox@GNCs could not only precisely target drugs to NSCLC cells but also effectively destroy H1299 cells by chemotherapy and PTT.^[66]^

In summary, due to their unique physical and chemical properties, gold nanomaterials have great potential in the field of tumor therapy; however, gold nanomaterials are prone to causing the aggregation of gold elements in tissue cells, and their long-term use may be toxic to the organism. Therefore, their metabolism and distribution in the organism, pharmacokinetic characteristics, safety, stability, and efficacy need to be further studied.

#### 4.2.4. Other inorganic nanomaterials

In addition to the above nanomaterials, other inorganic nanomaterials, such as silver nanoparticles and silica nanoparticles, are often used in tumor therapy. Silver nanoparticles play an important role in tumor therapy due to their good permeability, conductivity, and quantum size effect.^[67]^ When silver nanoparticles are applied to lung cancer A549 cells, the proliferation rate of the cells slows down with the increase in the concentration of silver nanoparticles.^[68]^ Silicon dioxide nanoparticles are commonly used nanomaterials in PDT and have the characteristics of easy modification, large pore size and specific surface area, and good biocompatibility. Moreover, they can also increase the production of oxygen in a single linear state (^1^O_2_).^[69]^ Zhan et al constructed magnetic and pH dual-targeted mesoporous silica nanoparticles that could be loaded with a higher amount of the photosensitizer Rose Bengal and highly aggregated in tumor cells, which could significantly reduce phototoxicity in normal tissues, increase the production of ^1^O_2_, and improve the efficacy of PDT.^[70]^

## 5. Advances in the study of NDDS in the treatment of lung cancer

### 5.1. NDDS and surgical treatment

Surgical resection of tumor tissue is the first line of treatment for lung cancer.^[71]^ In the process of surgical treatment, it is still challenging for clinicians to accurately determine the tumor boundaries and the need for lymph node dissection during surgery. Local recurrence and lymph node metastasis are important factors affecting the survival rate of lung cancer patients. In addition, with the development of imaging technology, more and more lung nodules are being detected. During the procedure, the surgeon can detect the location and size of pulmonary nodules by pleural retraction or manual palpation; however, when small nodules are located deep in the lung parenchyma, they may cause the pleura to be unaffected and prevent manual examination.^[72]^ Although preoperative CT-guided localization of pulmonary nodules (such as coils or hook wires) has been commonly used in clinics, this invasive method may lead to serious complications such as pneumothorax, hemothorax, and air embolism.^[73]^ Therefore, there is an urgent need for a noninvasive method to achieve rapid intraoperative identification and precise resection of tumor cells, thereby reducing tumor recurrence and improving the prognosis and survival of lung cancer patients.

Indocyanine green (ICG) can be used to track blood perfusion and identify tumor tissue during surgery. However, ICG lacks specificity and sensitivity for identifying tumor tissue and has a short circulation time in vivo.^[74]^ Carrying ICG on the surface of nanomaterials is expected to prolong the circulation time of ICG in vivo. Some researchers connected glycol ChNPs and ICG by conjugation. It was found that the cell viability was not affected by ICG-CNPs at a concentration of 200 μg/mL, and in the rabbit model injected with ICG-CNPs, a strong NIR light signal could be observed in the tumor tissues, which lasted for 96 hours. In contrast, no fluorescent signal was observed in the lung cancer tissues of the rabbit model with ICG injection alone. Combining white light and fluorescence images can clearly show the edge of the tumor and can guide tumor surgery under NIR fluorescence imaging.^[75]^ Similarly, Han et al designed a nanoplatform (PEG/MnCuDCNPs@GOx) that can be used for the early diagnosis and treatment of lung cancer. Due to the presence of Mn^2+^ and Cu^2+^, it can mediate the Fenton-like reaction in the acidic TME to exert chemodynamic therapy to eliminate cancer cells. The tumors were transplanted into mice for about 3 days, and PEG/MuCuDCNPs@GOx was injected through the tail vein. The NIR-IIb fluorescence signal detected a small tumor with a volume of about 1 cubic millimeter after 3 hours. Compared with the NIR-I signal, the NIR-IIb fluorescence signal is more sensitive, which can distinguish the tumor tissue from the normal tissue and guide the tumor resection.^[76]^

In conclusion, combining NDDS with fluorescent agents facilitates surgical tumor resection.^[77]^ However, this combination is challenging in clinical translation. First, the long-term toxicity of nanomaterials has not been fully demonstrated. Second, most of the studies of drug delivery systems combined with fluorescent agents have been cellular and animal experiments. Therefore, more clinical studies must be conducted in the future.

### 5.2. NDDS and chemotherapy

Chemotherapy, represented by cytotoxic drugs, is one of the most important treatments for lung cancer.^[78]^ At present, the most commonly used chemotherapeutic drugs are mainly PTX, platinum, and fluorouracil. However, these chemotherapeutic drugs usually lack targeting, and during the process of tumor chemotherapy, normal tissue cells will also be damaged, resulting in serious adverse reactions and making patients unable to tolerate long cycles of chemotherapy.^[79]^ In addition, tumor resistance and the poor water solubility of drugs also limit the effectiveness of chemotherapeutic drugs in clinical applications.^[80]^ NDDS can concentrate antitumor drugs in tumor tissues, prolong the half-life of drugs, and improve the solubility of hydrophobic drugs. For example, Pristimerin (PRI) has been shown to have antitumor effects, but its free form may cause serious toxic side effects. FA-modified nanomicelles (NMs) are loaded with PRI and PTX to construct PRI@FA-PEG-PTX (P@FPP) NMs. The results suggest that P@FPP NMs can achieve drug accumulation in tumor tissues by targeting the folate receptor, thereby alleviating the side effects of PRI. In addition, P@FPP NMs also significantly inhibit the growth of transplanted tumors in mice compared to PRI and PTX.^[81]^

Genetic alterations in tumor cells lead to abnormal cell proliferation, apoptosis resistance, and a shift in cellular metabolism to anaerobic glycolysis. These may result in the TME exhibiting acidosis, hypoxia, and oxidative stress.^[82]^ Based on the above TME properties, NDDS can take advantage of the special environment of the TME to promote targeted drug delivery. For example, pH-responsive nanoparticles are in a stable state in the neutral blood circulation. When entering the tumor tissue, its pH-responsive element deforms under the weakly acidic TME conditions to release the drug, realizing the targeted delivery of the nanodrug.^[83]^ Zhang et al designed a pH-dependent nanopolymer micelle (PPT/D(DMA)@DOX), which has good stability under an environment of pH 7.4. When PPT/D(DMA)@DOX changed from the physiological state (PH 7.4) to TME (PH 6.8), the negative charge on the surface of the micelles was transformed into a positive charge, which resulted in the easy uptake of the micelles by tumor cells. Upon entry into tumor cells, low pH in the endo/lysosome induces micelle degradation, which promotes DOX release.^[84]^

The development of resistance to chemotherapy is a major obstacle in the treatment of lung cancer. Cancer stem cells (CSCs) are considered to be one of the major causes of tumor recurrence, drug resistance, and metastasis.^[85]^ CSCs can express high levels of ABC transporter protein (ATP-binding cassette transporter), which improves tumor cell survival in chemotherapy.^[86]^ Moreover, CSCs have an active DNA repair capacity and are inherently resistant to chemotherapy.^[87]^ Therefore, targeting CSCs is a feasible strategy to avoid chemotherapy resistance. Increasing evidence suggests that chemotherapy can effectively kill cancer cells but cannot completely eliminate CSCs, and thus tumors are prone to drug resistance and recurrence. An anti-CD133-modified targeting system based on mesoporous silica nanoparticles can specifically target CSCs and induce apoptosis of CSCs under hypoxic conditions by blocking the HIF signaling pathway, thereby increasing tumor sensitivity to chemotherapy.^[88]^

In summary, NDDS can increase drug accumulation in tumor tissues, reduce the toxic side effects of chemotherapy through targeted drug delivery, and also show great potential for overcoming chemotherapy resistance. However, when developing stimulus-responsive nanosystems, premature drug release should be minimized. In addition, it is also very important to study the toxicity and metabolism of nanopreparations in vivo before clinical research.

### 5.3. NDDS and radiation therapy

Radiation therapy can cause DNA damage to cells or produce reactive oxygen species (ROS) to attack tumor cells. Radiotherapy consists of 2 types: external beam radiotherapy and internal radioisotope therapy.^[89]^ Due to the low absorption of radiation by tumor cells, a higher dose of irradiation is usually required to achieve therapeutic effects. This can cause serious damage to the surrounding normal tissues. In addition, the hypoxic microenvironment of tumor tissue also limits the effectiveness of radiation therapy.^[90]^ Therefore, the development of radiotherapy sensitizers can improve the sensitivity of tumors to radiotherapy, reduce the radiation dose, and decrease side effects.

Nanomaterials containing high atomic number elements (gold, bismuth, titanium, rare earth elements, etc) can catalyze the production of ROS.^[91]^ Various metallic nanoparticles are widely used to increase energy deposition at the tumor site, thereby enhancing the efficacy of radiation therapy. For example, radiation-sensitive bismuth selenide (Bi_2_ Se_3_) nanoparticles are loaded on adipose mesenchymal stem cells (AD-MSCs/Bi_2_ Se_3_). The accumulation of Bi_2_ Se_3_ NPs in tumors is increased 20-fold by AD-MSC-mediated targeted delivery. Radiosensitization and tumor growth inhibition are confirmed in an A549 tumor mouse model, which shows great potential for enhancing radiotherapy in lung cancer.^[92]^ In addition, albumin-modified gold nanoparticles (Alb-GNPs) are prepared by researchers. The sensitization enhancement ratio of Alb-GNPs is 1.432 compared to X-rays alone, which indicates that the complex had excellent radiosensitization. It is demonstrated by animal experiments that the survival of tumor-bearing mice treated with Alb-GNPs in combination with radiotherapy is longer than that of the control group.^[93]^ Another strategy to promote sensitization by radiotherapy is to alter the tumor hypoxic microenvironment. Song et al loaded catalase inside TaOx hollow nanoshells, and the complex could decompose endogenous H_2_O_2_ to provide oxygen in the TME, thus ameliorating the hypoxia of the TME. In addition, TaOx nanoshells were able to significantly improve the efficacy of radiotherapy by depositing radiation energy into tumor tissues and increasing radiation-induced DNA damage.^[94]^

In addition to the sensitization of radiotherapy, the protection of normal tissues in radiotherapy is also a key research direction of NDDS. Inorganic metal nanomaterials play an important role in radiotherapy protection due to their excellent catalytic properties. Inorganic nanomaterials such as Ce and Hf can remove ROS generated by radiotherapy in normal tissues, thus possibly reducing the incidence of radiation pneumonitis. It is found that the nanoparticles are prepared by whey protein-loaded hafnium dioxide, which exhibit radiotherapeutic protection in both cell and animal experiments. The radioprotective effect is determined by the unfolded ligand and the Hf(0)/Hf(IV) redox pair. By adjusting the synthesis ratio of whey protein and hafnium dioxide, it can be targeted to normal tissues that are more sensitive to radiation, thus protecting normal tissues from radiotherapy.^[95]^

As mentioned above, the potential and value of NDDS in radiotherapy is mainly as a sensitizer to improve radiotherapy efficacy. Although the application of NDDS in lung cancer radiotherapy can improve the therapeutic efficacy and safety, the damage to normal cells caused by radiotherapy is still unavoidable, which may lead to the decline of immune function and the possibility of myelosuppression. In the future, we should continue to research and develop new nanomedicines to reduce the impact on normal cells and the toxic side effects of treatment.

### 5.4. NDDS and immunotherapy

Immunotherapy is a therapeutic method to inhibit and kill tumors by actively or passively causing the body to produce a tumor-specific immune response. However, unpredictable therapeutic efficacy, treatment-related toxicity, and adaptive immune resistance are still important issues that need to be addressed in immunotherapy.^[96]^ Several nanomaterial-based strategies, such as the promotion of tumor immunogenic death, combined with immune checkpoint blockade immunotherapy, cancer vaccines, overdose immunotherapy, and immune microenvironment modulation, are promising for improving tumor immunotherapy.

Immune checkpoint inhibitors (ICIs) of programmed cell death receptor 1 (PD-1) and its ligand (PD-L1) are considered to be the most commonly used drugs in lung cancer immunotherapy. Conventional ICIs have low immunogenicity, weak targeting, and susceptibility to drug resistance, which limits their therapeutic efficacy. The efficiency of anti-PD-1 monoclonal antibody injection alone is only 20% to 30%.^[97]^ NDDS can effectively improve the blocking efficiency of ICIs and can also synergize the treatment of tumors by combining with other therapeutic approaches. A nanodrug (SGT-53) can restore the function of the p53 gene, thereby enhancing anti-PD-1 inhibition of lung cancer growth and prolonging the survival of tumor-bearing animals. In addition, SGT-53 also restores an effective immune response to lung cancer cells by decreasing M2-type macrophages and increasing cytotoxic T-cell activity.^[98]^ Integrin β3 is found to be upregulated in patients with spinal cord metastases from NSCLC; most of these patients are insensitive to ICIs. Zhou et al^[99]^ prepared a novel nanoparticle (ZnPP@MSN-RGDyK) that precisely targets integrin β3, thereby inhibiting the expression of PD-L1 and showing excellent immunotherapeutic effects in a mouse model.

In addition to immune checkpoint therapy, drug development targeting immune cells in the tumor immune microenvironment, such as tumor-associated macrophages (TAMs) and regulatory cells (Tregs), is also progressing. TAMs are mainly classified into M1 and M2 types, and M1 macrophages can kill tumors and promote antitumor immune responses. M2 macrophages inhibit antitumor immunity and promote tumor development.^[100]^ Therefore, TAMs are one of the targets of tumor immunotherapy. It is found that a liposomal nanoparticle containing cyclic dinucleotides can reprogram TAMs from the M2 type to the M1 phenotype, resulting in effective tumor antigen presentation and increased anticancer immune responses.^[101]^ Furthermore, an increase in Tregs in the TME is indicative of tumor progression, and Tregs are able to influence the antitumor responses of effector T cells through a variety of mechanisms. It is found that decreasing the number of Tregs in the TME can enhance the antitumor immune response.^[102,103]^ A photo-activated silica-phthalocyanine dye-conjugated anti-CD25 antibody nanodrug eliminates Tregs in a Lewis lung cancer model. When NIR light is applied, NIR-PIT targeting CD25 induces selective depletion of regulatory T cells, which leads to the activation of CD8^+^ T and NK cells and restores the antitumor effect of local immunity.^[104]^

Overall, immunotherapy provides a new therapeutic approach for lung cancer patients; however, the therapeutic efficacy is severely affected due to the presence of the TME. NDDS can enhance the immunotherapeutic response by modulating immune cells in the TME, which shows great potential for the future outlook of lung cancer treatment.

### 5.5. NDDS and targeted therapy

Molecularly targeted therapy has been a major breakthrough in the treatment of malignant tumors in recent years, significantly prolonging the survival time of patients. Targeting mutated molecular targets, such as EGFR and anaplastic lymphoma kinase, can effectively inhibit tumor cell proliferation. Currently, tyrosine kinase inhibitors (such as GEB and erlotinib) have been widely used in clinical treatment.^[105]^ However, poor water solubility, slow absorption, and gastrointestinal and other adverse effects of oral tablets often affect the efficacy of the drug and its distribution in the body. In addition, drug resistance often occurs in some patients after treatment due to off-target side effects, secondary mutations in EGFR (T790M), and activation of alternative pathways.^[106]^ Various nanotechnology-based delivery systems can improve drug utilization and stability, prolong blood circulation time in vivo, and increase drug concentration in tumor tissues.

In order to improve the aqueous solubility of the drug and prolong the circulation time in the body, Wang et al encapsulated GEB in lactic acid-ethanolic acid copolymerized nanoparticles of carboxymethyl chitosan, which improved the solubility and stability of GEB and prolonged the time of drug release. The bioavailability of the composite nanoparticles was increased by approximately 1.6-fold compared to GEB alone.^[107]^ However, uncontrolled drug release leads to wide distribution of the drug in the body and affects the efficacy of molecularly targeted therapies. Lin et al reported a NDDS (MSNs@Ag@Geb-FA) combining photothermal and molecularly targeted therapies, which consisted of FA targeting the folate receptor, GEB, photothermite (Ag), and mesoporous silica (SiO_2_) heterostructures. Under FA mediation, MSNs@Ag@Geb-FA could target tumor cells. In the weak acid environment of TME, it could specifically promote the release of Ag and GEB in cells.^[108]^

In lung adenocarcinoma patients with EGFR gene mutations, tumor progression is controlled in most of the patients after treatment with EGFR-TKIs. However, as the number of medications increased, some patients developed drug resistance.^[109]^ To address the resistance of TKIs, Wang et al used cetuximab-capped mesoporous silica nanoparticles (MP-SiO_2_ NPs) loaded with GEB to address GEB resistance. It was found that high levels of glutathione in the GEB-resistant cell line (PC9-DR) induced an effective release of GEB, which significantly inhibited the proliferation of PC9-DR cells. Thus, NDDS have great potential for overcoming drug resistance.^[110]^

To summarize, NDDS can improve drug solubility and stability and effectively target drugs to lung tumor cells. In addition, NDDS can also enhance the efficacy of drugs and reduce the drug resistance of tumor cells. It shows great promise in the treatment of advanced lung cancer. Table 2 summarizes the research progress of different NDDS in lung cancer treatment.^[111–117]^

**Table 2:** Advances in nanodrug delivery systems in lung cancer therapy.

	Nanocarriers	Cancer type	Tumor model	Results	References
Cisplatin and docetaxel	Protein-lipid hybrid nanoparticle	LC	Nude mice	By improving the uptake efficiency of tumor cells, the accumulation of drugs in the tumor is increased, thus inhibiting the growth of the tumor	^[111]^
Docetaxel	Chitosan nanoparticles	LC	Wistar rats	Enhancing cytotoxicity of doxorubicin on A549 lung cancer cells	^[112]^
Genistein	Nanoparticle	NSCLC	Mice	Improving radiation protection of normal lung tissue	^[113]^
Gadolinium	Nanoparticle	NSCLC	H1299 mouse xenograft model	Enhancing the sensitivity of cancer cells to radiation and promoting the apoptosis of cancer cells	^[114]^
Cisplatin	Nanoparticle	—	Lewis lung carcinoma tumor model	Promoting therapeutic effects of anti-PD-1/PD-L1 blockade	^[115]^
Volasertib and PD-L1 antibody	Nanoparticle	NSCLC	metastatic lung tumor model	Enhancing the effect of PD-L1 inhibitors	^[116]^
Gefitinib	Black phosphorus nanoparticle	NSCLC	—	Promoting delivery of gefitinib to tumor tissue and prolonging its time in the tumor	^[117]^

LC = lung cancer, NSCLC = non-small cell lung cancer, PD-1 = programmed cell death receptor 1, PD-L1 = programmed cell death ligand 1.

## 6. Conclusions and future perspectives

Lung cancer is currently the malignant tumor with the highest mortality rate. Although there have been tremendous advances in the treatment of lung cancer in the last decade, the mortality rate associated with lung cancer remains high. This article summarizes the current treatment strategies and shortcomings of lung cancer in order to find new treatments to improve the survival time of lung cancer patients. Nanocarrier-based drug delivery systems can deliver drugs to tumor tissues in a targeted manner and effectively inhibit the growth of cancer cells. Recent findings suggest that nanotechnology-based approaches in combination with surgical treatment, chemotherapy, radiation therapy, immunotherapy, and molecularly targeted therapy show great potential in the treatment of lung cancer.

Despite the great progress of NDDS in medical oncology research, the clinical translation of NDDS still faces many challenges. First, more in vivo studies are needed to confirm and evaluate the safety and toxicity of NDDS before they are used in clinical trials in the future. Second, some clinical trials of NDDS for lung cancer treatment have been terminated because the therapeutic effect was not as expected, so how to improve the tumor-targeting ability and therapeutic effect of NDDS in the human body needs to be further investigated; in addition, the production process of NDDS is more complicated and the storage conditions are more demanding, so the actual application in the clinic needs to gradually improve the production process and simplify the storage and transportation conditions. Finally, how to scientifically select and match the therapeutic drugs with NDDS to achieve the best therapeutic effect and minimize the adverse effects. It is believed that in the near future, with the joint efforts of scientists in the fields of material science and clinical medicine, the difficulties in the clinical application of NDDS will be solved, and NDDS will play an important role in the treatment of lung cancer.

## Author contributions

**Conceptualization:** Jiang Fu, Li Yu, Zixu Wang.

**Data curation:** Jiang Fu, Song Zhang.

**Resources:** Jiang Fu, Haining Zhou.

**Software:** Jiang Fu.

**Validation:** Jiang Fu, Song Zhang.

**Visualization:** Jiang Fu.

**Writing – original draft:** Jiang Fu, Li Yu.

**Writing – review & editing:** Jiang Fu, Li Yu.

**Formal analysis:** Li Yu, Zixu Wang.

**Methodology:** Haoyu Chen.

**Funding acquisition:** Haining Zhou.

**Project administration:** Haining Zhou.

**Supervision:** Haining Zhou.
